# Spatiotemporal Multivariate Weather Prediction Network Based on CNN-Transformer

**DOI:** 10.3390/s24237837

**Published:** 2024-12-07

**Authors:** Ruowu Wu, Yandan Liang, Lianlei Lin, Zongwei Zhang

**Affiliations:** 1 State Key Laboratory of Complex Electromagnetic Environment Effects on Electronics and Information System, Zhengzhou 450003, China; 2 School of Astronautics, Harbin Institute of Technology, Harbin 150001, China; 2022112465@stu.hit.edu.cn; 3 School of Electronics and Information Engineering, Harbin Institute of Technology, Harbin 150001, China; linlianlei@hit.edu.cn (L.L.); zongwei_cumt@163.com (Z.Z.)

**Keywords:** digital earth, spatiotemporal series prediction, weather prediction, convolutional neural networks, transformer

## Abstract

Weather prediction is of great significance for human daily production activities, global extreme climate prediction, and environmental protection of the Earth. However, the existing data-based weather prediction methods cannot adequately capture the spatial and temporal evolution characteristics of the target region, which makes it difficult for the existing methods to meet practical application requirements in terms of efficiency and accuracy. Changes in weather involve both strongly correlated spatial and temporal continuation relationships, and at the same time, the variables interact with each other, so capturing the dynamic correlations among space, time, and variables is particularly important for accurate weather prediction. Therefore, we designed a spatiotemporal coupled prediction network based on convolution and Transformer for weather prediction from the perspective of multivariate spatiotemporal fields. First, we designed a spatial attention encoder-decoder to comprehensively explore spatial representations for extracting and reconstructing spatial features. Then, we designed a multi-scale spatiotemporal evolution module to obtain the spatiotemporal evolution patterns of weather using inter- and intra-frame computations. After that, in order to ensure that the model has better prediction ability for global and local hotspot areas, we designed a composite loss function based on MSE and SSIM to focus on the global and structural distribution of weather to achieve more accurate multivariate weather prediction. Finally, we demonstrated the excellent effect of STWPM in multivariate spatiotemporal field weather prediction by comprehensively evaluating the proposed algorithm with classical algorithms on the ERA5 dataset in a global region.

## 1. Introduction

Digital Earth [1] can help scientists and decision-makers analyze and understand complex Earth systems in a more comprehensive and coordinated manner. Within the framework of Digital Earth, climate prediction is of great significance in supporting scientific research and decision-making [2], enhancing the ability to cope with natural disasters [3], addressing global climate change [4,5], and ultimately improving agricultural production [6,7]. For example, climate prediction models that accurately predict the path of hurricanes can warn coastal residents and organize mass evacuations in time to reduce casualties. For instance, during Hurricane Harvey in 2017 and Hurricane Michael in 2018, accurate forecasts led to the early evacuation of hundreds of thousands of people, preventing potential loss of life [8]. Through accurate weather forecasting, we can better cope with the impacts of climate change, protect lives and property, safeguard economic stability, maintain ecological balance, and promote global climate governance.

With the development of meteorological science, forecasting methods are evolving and diversifying. In modern weather forecasting, numerical weather prediction [9] and data-driven methods [10], two core technology paths, rely on the atmospheric physics model and big data analysis to provide different perspectives of weather prediction capabilities. The core of numerical weather prediction lies in acquiring accurate initial conditions [11], and initial condition errors can accumulate in the model over time, leading to a decrease in forecast accuracy, which is especially noticeable in long-term prediction. Meanwhile, some complex small-scale physical processes (e.g., cloud physics, turbulence) must be parameterized, which may introduce errors [12]. Moreover, numerical weather prediction is highly demanding in terms of computational resource requirement [13]. These limitations diminish the applicability of numerical models in weather prediction.

Data-driven models can be categorized into machine learning and deep learning models, both of which are techniques for learning patterns and regularities from data. Data-driven methods have become increasingly crucial in climate prediction in recent years. Data-driven models can automatically extract complex patterns from massive amounts of meteorological data, opening up new possibilities for more accurate climate predictions. Machine learning has significantly improved the accuracy and reliability of climate prediction in several aspects, including climate pattern recognition and classification [14], forecasting and warning of weather events [15], multi-model integration and optimization [16], uncertainty quantification, and risk assessment [17,18]. These technological advances are pushing the frontiers of climate science research and providing solid technological support to combat global climate change. Support vector machines (SVM), artificial neural networks (ANN), and K-nearest neighbors (KNN) were used to develop drought prediction models in Pakistan [19]. Experiments have shown that SVM-based algorithms can better capture the temporal and spatial characteristics of drought in Pakistan. Ref. [20] demonstrated better results by stacking multilayer perceptron and random forest (MLP-RF) to predict the lake surface water temperature with day-by-day air temperature as an exogenous input variable and applying a Bayesian optimization procedure to tune the hyperparameters. However, machine learning models are susceptible to the quality and integrity of the input data. Noise, missing values, or bias in the data may significantly affect the model performance and prediction results [21]. Traditional machine learning models (e.g., support vector machines and random forests) usually require manual feature selection and engineering design. This process is time-consuming and requires deep knowledge from domain experts, limiting the automation and adaptability of the model. Additionally, the generalization capability of new data may be affected [22]. In cases of improper feature selection, overfitting problems may significantly affect the model’s predictive ability.

Deep learning can extract high-level features and complex patterns from raw data, reducing the reliance on manual feature engineering. In addition, deep learning models have more robust nonlinear modeling capabilities and can handle complex, high-dimensional datasets, providing more accurate predictions [23]. Ref. [24] used long short-term memory (LSTM) and its variants to test the accuracy of data-driven models in predicting extreme events. The experiments demonstrate that LSTM networks remain relatively accurate in predicting extreme events, even if the extreme events are not included in the training set. Ref. [25] proposed an hour-by-hour rainfall-runoff estimation model based on LSTM and seq2seq structure. Ref. [26] constructed an LSTM-based hybrid model, LSTM-CM, for drought prediction. Compared with LSTM-SA and GloSea5 (GS5), LSTM-CM can accurately detect drought events with less prediction uncertainty. However, climate data not only includes time series, but also involves complex connections in the spatial dimension. LSTM mainly focuses on the one-dimensional dependence of time-series data, and thus, LSTM alone cannot adequately capture the spatial dependence and heterogeneity in meteorological data. Ref. [27] developed two hybrid LSTM-based models for monthly flow and rainfall prediction at multiple observation sites. One of these models is the wavelet-transformed trous algorithm WLSTM, which applies a wavelet-transformed trous algorithm for sequence decomposition. The other is convolutional LSTM (CLSTM), coupling convolutional neural networks to extract temporal features. The results reflect the superiority of convolutional LSTM for climate prediction, as convolution can better extract the spatial connections in the data.

To improve the limitations in spatial feature modeling and spatial-temporal interaction information extraction and to enhance the model’s ability to process spatial and temporal information in an integrated manner, Ref. [28] proposed a deeply coupled model of convolution and LSTM, ConvLSTM, which has achieved good results in large-area rainfall prediction. Ref. [29] studied ConvLSTM and spatiotemporal ConvLSTM and found that ConvLSTM and spatiotemporal ConvLSTM have the best prediction performance when the input length is set to 1, and the prediction performance decreases as the input length increases. Ref. [30] investigated the effectiveness of three deep learning methods, namely, LSTM, convolutional neural network (CNN), and ConvLSTM, in short-term flow prediction in river basins. It was found that ConvLSTM works better in large river basins. Ref. [31] integrated CNN with LSTM and a gated recurrent unit (GRU) to achieve the best results in one-day-ahead air temperature prediction. Although the above methods have improved the accuracy of climate prediction by introducing 2D convolution, their application still has many limitations. For example, the practical receptive fields of CNN operators are relatively small, limiting the model’s ability to capture large-scale information [32,33]. The features extracted by shallow convolutional networks are usually low-level and localized. Such features show a deficiency in expressing the global structure of the data or high-level semantic information [34].

For complex spatial-temporal meteorological data, global features are essential information for understanding the overall data distribution, long-term trends, and large-scale patterns. Self-attentive mechanisms [35,36] can capture long-range dependencies in the series and effectively model global information. Since its introduction, Transformer [37] has rapidly evolved and achieved remarkable success in several domains [38,39,40], and its self-attention mechanism as a core component effectively captures global dependencies. The Transformer model has also been introduced to computer vision, resulting in the Vision Transformer (ViT) and other new models [41]. ViT utilizes the Transformer to process image blocks directly instead of pixels, showing performance that competes with or surpasses that of CNNs. The spatiotemporal hybrid convolutional attention network (StHCFormer) utilizes separate attention to extract features in time and space separately and with convolution to obtain local features, but the iterative prediction approach makes the accuracy of its long-step prediction limited [42]. The computational complexity of the self-attention mechanism is $O(n^{2})$, where *n* is the length of the input sequence. This means that the computational demand grows by a square as the sequence length increases, leading to high memory consumption and computational resource demand when processing long sequence data [37]. It also performs poorly in terms of memory footprint [43]. Moreover, the self-attention mechanism emphasizes the extraction of global features, but cannot handle fine-grained local features [44].

According to the limitations of the existing weather prediction methods and the specificity of weather prediction, this study proposes a spatiotemporal coupled network based on CNN-Transformer to predict future weather, named the spatiotemporal weather prediction model (STWPM). Our main contributions can be summarized as:

1. We propose a new multivariate weather prediction model named STWPM. It uses self-attention to construct a spatial encoder-decoder to extract and reconstruct the weather spatial features. The model avoids error accumulation by directly outputting multi-step future weather through the decoder.

2. We design a new multi-scale spatiotemporal evolution module to capture the spatiotemporal evolution pattern of weather by performing multi-scale feature extraction from both intra- and inter-frame perspectives.

3. We introduce mean square error and structure similarity index measure composite loss functions to enhance weather prediction. By considering global and structural aspects together, it can help the forecast network adapt to complex changes in weather data.

4. We compare STWPM with other methods on publicly available datasets. The results demonstrate that STWPM can successfully offer accurate and efficient weather forecasting.

## 2. Methodology

### 2.1. Problem Definition

Spatiotemporal weather fields are usually divided into grids based on spatial information. Each grid is an observation point for multivariate weather, and each cycle contains a set of multivariate values. All grid areas form a C×H×W matrix $D_{t}$ representing a set of weather values at a specific time *t*, where *W* and *H* correspond to the number of grid areas along latitude and longitude, respectively, and *C* denotes the number of variables. All matrices in a region’s historical data record combine to generate the spatiotemporal fields series $\left\{{D_{1}}^{\left(\alpha ,\beta \right)},{D_{2}}^{\left(\alpha ,\beta \right)},...,{D_{t}}^{\left(\alpha ,\beta \right)},{D_{t}}^{\left(\alpha ,\beta \right)}\in R^{T\times 2\times H\times W}\right\}$ if the two variables (α,β) are taken into account.

In practice, we usually try to learn from a period of historical weather records and use what we learn to predict future weather conditions. The weather forecasting work we carry out follows this principle. Our time point interval is selected to be 6 h since while predicting the weather, we often assume a 6 h window or shorter, wherein the weather will not vary significantly. In this way, there are 4 time points for each day: 0:00, 6:00, 12:00, and 18:00. For a certain set area, given *u* multivariate histories $\left\{{D_{1}}^{\left(\alpha ,\beta \right)},{D_{2}}^{\left(\alpha ,\beta \right)},...,{D_{u}}^{\left(\alpha ,\beta \right)}\right\}$, the future *v* weather data are inferred from the *u* histories, and the formula is expressed as:

(1) $$D_{u+1}^{\left(\alpha ,\beta \right)},...,D_{u+v}^{\left(\alpha ,\beta \right)}=g\left(D_{1}^{\left(\alpha ,\beta \right)},D_{2}^{\left(\alpha ,\beta \right)},...,D_{u}^{\left(\alpha ,\beta \right)}\right)$$

where *g* denotes the prediction network. For example, when u=28 and v=20, according to the previously set 6 h point taking law, it means that we forecast the following 5 days (α,β) using the past 7 days (α,β).

### 2.2. STWPM

**Figure 1:**
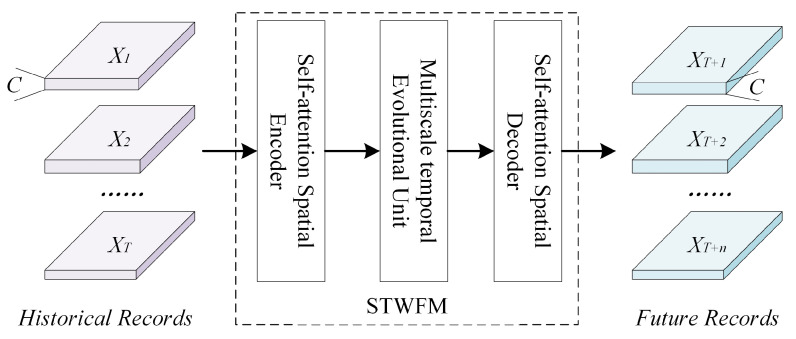
Overall network framework.

The structure of the prediction network in this paper is shown in Figure 1. It mainly consists of inputs and outputs, a self-attention spatial coding decoder, and a multi-scale time evolution unit. The input feature is $I\in R^{B\times T\times C\times H\times W}$, where *B* denotes batch size, and *T* denotes time series length. First, we shape *I* into $I_{s}\in R^{BT\times C\times H\times W}$ and feed it into the spatial encoder for spatial feature extraction. After that, we shape the feature into $I_{t}\in R^{B\times TC\times H\times W}$ and send it to the time unit for evolution to obtain the spatial features of the weather in the future moment, and finally, we use the spatial decoder to reconstruct the distribution of the weather in said future moment.

**Figure 2:**
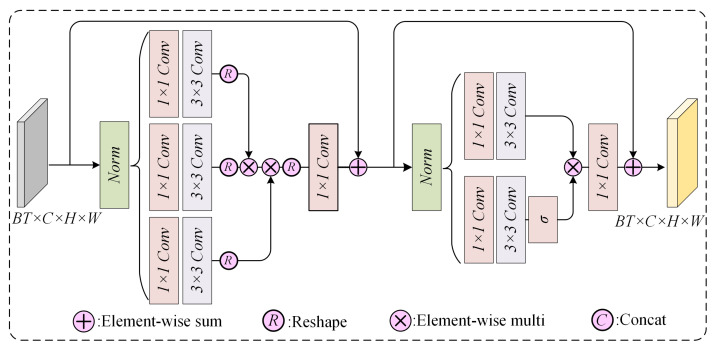
Transformer-based encoder-decoder structure.

Self-attention spatial encoder-decoder: The self-attention mechanism calculates dynamic association weights between all positions in a sequence, enabling each position to interact with global information and thereby flexibly capture and extract global features [37,41]. To extract the spatial dependence of the meteorological field, we use Transformers to construct a self-attention spatial encoder to extract features. Considering the computational efficiency and balance performance, we use the LT block [33] as the basic unit of the encoder and decoder, and its network structure is shown in Figure 2.

Taking the bivariate input $I_{s}\in R^{BT\times 2\times H\times W}$ of the spatial encoder as an example, after normalization, *Q* (query), *K* (key), and *V* (vector) can be obtained using 1 × 1 convolution and 3 × 3 deep convolution, which can be expressed in the following equation:

(2) $$Q=W_{b}^{Q}W_{a}^{Q}I_{s}$$

(3) $$K=W_{b}^{K}W_{a}^{K}I_{s}$$

(4) $$V=W_{b}^{V}W_{a}^{V}I_{s}$$

where *Q*, *K*, and *V* are vectors obtained by convolutionally transforming the input features, *Q* and *K* determine the degree of attention, and *V* denotes the weighted aggregated features. Their dimensions are BT×C×H×W. $W_{a}$ and $W_{b}$ denote 1 × 1 unbiased convolution and 3 × 3 unbiased depth convolution, respectively. Next, we reshape the query and key projections and make dot-product interaction to obtain attention map *A*. The following equation can summarize the process of attention calculation:

(5) $$A\left(\hat{Q},\hat{K},\hat{V}\right)=\hat{V}\cdot \sigma \left(\hat{K}\times \hat{Q}/\alpha \right)$$

(6) $${\hat{I}}_{A}=W_{a}A+I_{A}$$

where ${\hat{I}}_{A}$ and $I_{A}$ are the input and output features of the attention module. Here, α is a learnable scaling parameter used to control the size of the dot product of *K* and *Q* before applying the softmax function. Like the traditional multi-head self-attention model, we divide the channels into “heads” and learn individual attention maps in parallel. The following equation can represent the regular feed-forward network:

(7) $$FF\left({\hat{I}}_{A}\right)=\sigma \left(W_{a}^{u}W_{b}^{u}\left({\hat{I}}_{A}\right)\right)⊙W_{a}^{d}W_{b}^{d}\left({\hat{I}}_{A}\right)$$

(8) $${\hat{I}}_{AF}=W_{a}\;FF\left({\hat{I}}_{A}\right)+{\hat{I}}_{A}$$

where ⊙ denotes the elemental multiplication and σ denotes the Sigmoid activation function.

**Figure 3:**
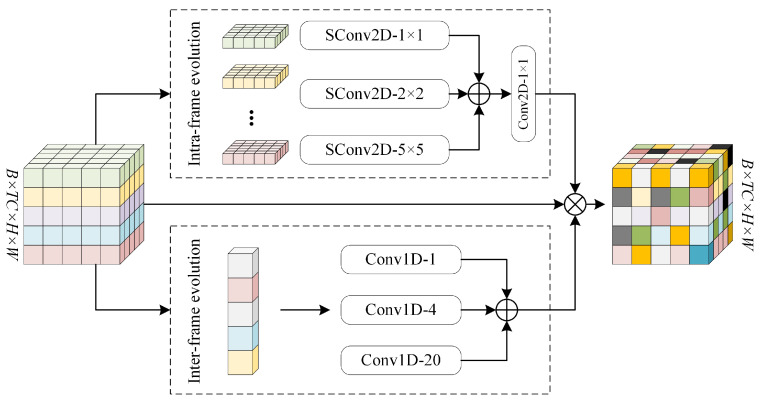
Multiscale spatiotemporal evolution module. SConv represents depth separation convolution, and Conv1D represents one-dimensional convolution.

Multiscale temporal evolution unit: We designed the multiscale time-series evolution module to obtain the spatiotemporal evolution law of meteorological features. The multiscale temporal evolution module contains two branches: the intra-frame evolution branch and the inter-frame evolution branch. As shown in Figure 3, in the intra-frame evolution branch, we extract the intra-frame evolution law of the feature channel by depth-separated convolution in aggregating the features by 1 × 1 convolution. Convolution excels at extracting local features by leveraging small receptive fields to capture spatial patterns such as edges, textures, and shapes, making it highly effective for analyzing localized image regions. Depthwise separable convolution builds on this advantage by splitting the standard convolution into depthwise convolution for spatial feature extraction within each channel and pointwise convolution for cross-channel information integration, thereby enhancing computational efficiency while maintaining precise local feature extraction. Assuming the input feature is $I_{t}\in R^{B\times TC\times H\times W}$, then the intra-frame evolution branch can be represented as:

(9) $$I_{inter}=W_{1\times 1}\left[DW_{1}\left(I_{t}\right)+DW_{3}\left(I_{t}\right)+DW_{5}\left(I_{t}\right)\right]$$

where $DW_{n}$ denotes a depth-separated convolution with convolution kernel size *n*, and $W_{1\times 1}$ denotes a 1×1 two-dimensional convolution.

In the inter-frame evolution branch, we first use adaptive pooling to process the input features $I_{t}\in R^{B\times TC\times H\times W}$ to obtain the 1D features ${I_{t}}^{\prime }$. To obtain the feature dependencies at different time scales, we use a multi-scale 1D convolutional group for feature extraction. Afterward, the weights between different nodes are normalized using a Sigmoid function, which ensures a balanced and meaningful representation of the inter-sequence relationships. The following equation can represent the inter-frame evolutionary branch:

(10) $${I_{t}}^{\prime }=Adp\left(I_{t}\right)$$

(11) $$I_{intra}=W_{1-1}\left({I_{t}}^{\prime }\right)+W_{1-4}\left({I_{t}}^{\prime }\right)+W_{1-20}\left({I_{t}}^{\prime }\right)$$

where $W_{1-n}$ denotes a one-dimensional convolution with convolution size *n*, and Adp denotes adaptive pooling. Finally, we avoid spatiotemporal feature loss by residual initial feature linking to obtain the final output of the multiscale temporal evolution module:

(12) $$I_{t\_o}=I_{inter}*I_{intra}*I_{t}$$

### 2.3. Loss Function

Weather is spatially continuous and does not change abruptly easily within a certain distance. However, the resolution of the data we acquire creates a more significant potential for sudden weather changes. The stability and fluctuation of the weather field will be reflected in the spatial manifestation as the distribution of low-frequency and high-frequency information. Fitting this structural distribution of high and low-frequency coexistence is difficult using only the mean square error (MSE) loss function. To adapt to the unpredictability of the meteorological field and enable our model to better capture the weather evolution patterns, we introduce the structural loss structure similarity index measure (SSIM) based on the global MSE, which is a commonly used loss function in the field of data generation, including image generation, spatiotemporal data prediction, etc. Here, we use $I_{P}$ and $I_{G}$ to denote the predicted and true values. Thus, we can obtain the MSE loss as:

(13) $$L_{Glo}={∥I_{P}-I_{G}∥}_{F}^{2}$$

In the image field, people often use SSIM [Appendix A] to evaluate the goodness of image recovery. SSIM measures image reconstruction based on three aspects: brightness similarity, contrast similarity, and structure similarity. Each frame of meteorological spatiotemporal data can be treated as a picture, and the meteorology distribution structure is equivalent to an image’s structure. Therefore, we introduce the SSIM metrics from the image domain into our weather prediction loss to assist the network in better fitting the spatial distribution of weather factors. The following equation can express the SSIM loss:

(14) $$L_{SSIM}=1-SSIM\left[I_{P},I_{G}\right]$$

where SSIM denotes the SSIM calculation process. Thus, the mixing loss in this paper can be expressed as:

(15) $$L=\omega L_{Glo}+\left(1-\omega \right)L_{SSIM}$$

where ω denotes the balancing parameter, which is used to balance the MSE loss and the SSIM loss.

## 3. Experiments

### 3.1. Dataset

In our experiments, we used the ERA5 reanalysis dataset [45] for training and testing to evaluate the effectiveness of the proposed model. The raw data contain daily data from between 1979 and 2018 with a temporal resolution of 1 h and a spatial resolution of 5.625° × 5.625°. The data from 1979–2016 were used for training and validation, and the data from 2017–2018 were employed for testing. In addition, we chose the northeastern region of China for the prediction experiment in a small-scale region. The experimental area is [36° N–52° N, 119° E–135° E], and the spatial resolution is 0.25° × 0.25°. We chose T850 temperature field data and Z500 geopotential field data to evaluate the STWPM model in this paper. We usually use the pressure in hundreds of pascals as the vertical coordinate in meteorological data prediction, which can make meteorological data more general and facilitate the study of meteorological changes on a global scale. The pressure at sea level is about 1000 hPa and decreases exponentially with altitude. The pressure potential at 500 hPa, often abbreviated Z500, is a commonly used variable to encode the weather-scale pressure distribution. It is a standard validation variable for most mid-range NWP models. Temperature at 850 hPa is usually abbreviated as T850. Compared to ground temperatures, T850 is less affected by daily variations, providing broader information on temperature trends.

### 3.2. Evaluation Metrics

In this paper, we use the metrics in [46] to evaluate the effectiveness of weather prediction, including RMSE, MSE, and ACC.

(16) $$RMSE=\frac{1}{N_{predictions}}\sum_{i}^{N_{predictions}}\sqrt{\frac{1}{N_{lat}N_{lon}}\sum_{j}^{N_{lat}}\sum_{k}^{N_{lon}}L\left(j\right){\left(f_{i,j,k}-t_{i,j,k}\right)}^{2}}$$

(17) $$MAE=\frac{1}{N_{predictions}}\sum_{i}^{N_{predictions}}\frac{1}{N_{lat}N_{lon}}\sum_{j}^{N_{lat}}\sum_{k}^{N_{lon}}L\left(j\right)∥f_{i,j,k}-t_{i,j,k}∥$$

(18) $$ACC=\frac{\sum_{i,j,k}L\left(j\right)f_{i,j,k}^{\prime }t_{i,j,k}^{\prime }}{\sqrt{\left(\sum_{i,j,k}L\left(j\right)f_{i,j,k}^{\prime 2}\right)\left(\sum_{i,j,k}L\left(j\right)t_{i,j,k}^{\prime 2}\right)}}$$

(19) $$L\left(j\right)=\frac{\cos(lat(j))}{\frac{1}{N_{lat}}\sum_{0}^{N_{lat}}\cos(lat(j))}$$

where *f* is the model’s predicted value and *t* is the true value of the ERA5 dataset. L(j) is the latitudinal weighting factor at latitude *j*, and the symbol ′ denotes the difference from climatology. Here, the climatology is defined as $climatology_{j,k}=\frac{1}{N_{time}}\sum t_{j,k}$.

### 3.3. Results Analysis

#### 3.3.1. Overall Performance Analysis

We tested the global weather data prediction results with lead times of 3 and 5 days to evaluate the performance of the STWPM in meteorological spatiotemporal data prediction. We conducted comparative experiments with several commonly used baseline models in [47,48]. In this paper, three evaluation indexes, RMSE, MAE, and ACC, are used to evaluate the prediction accuracy of meteorological data models, which comprehensively measure the performance of the models from different perspectives. RMSE is well suited to assessing model performance in extreme situations, helping to identify if the model has large errors for extreme weather events. MAE, suitable for assessing the model’s regular performance, reflects a smoother error case in the overall prediction. ACC is particularly applicable to assessing the model’s ability to predict meteorological anomalies or extreme weather events. The combined use of RMSE, MAE, and ACC can fully evaluate the accuracy of the meteorological models, which not only focuses on the overall prediction performance but also measures the model’s ability to deal with significant errors and climate anomalies, making the evaluation more scientific and complete. Table 1, Table 2 and Table 3 summarize the evaluation results of the models in terms of RMSE, MAE, and ACC.

The persistence model directly uses the current observation data as the future prediction value. It performs better in short-term prediction with slow and smooth weather changes but has lower prediction accuracy for unexpected weather events. The climatology model is suitable for long-term prediction. It reflects the average behavior of the meteorological variables well, but is unable to cope with extreme weather events. Linear regression (direct) ignores nonlinear features in meteorological data, resulting in lower prediction accuracy. Direct linear regression is susceptible to abnormal meteorological events (e.g., extreme weather), and these anomalies can lead to shifts in the model weights, affecting the overall prediction results. Linear regression (iterative) approaches the optimal solution step by step by constantly updating the model parameters. However, in the case of large data volumes or high feature dimensions, the convergence speed may be slower, and the consumption of computational resources is higher. Meanwhile, this method is still based on linear assumptions and fails to fully capture the weather system’s nonlinear features. Meteorological data have nonlinear solid characteristics. Compared with linear regression, CNN models can deal with complex nonlinear relationships in meteorological data. Also, CNNs are better at handling meteorological data with spatial features and can efficiently capture data’s local spatial features through convolution operation. CNN (direct) directly inputs meteorological data and outputs the predicted values once through a trained CNN model; its computation is one-time and does not involve iterative updating, so the long-time dynamic connection is neglected. CNN (iterative) gradually predicts the future meteorological state through iterative updating, which can better track temporal dimensions compared to CNN (direct). Meanwhile, CNN (iterative), using the gradual iterative way to expand the prediction time window step by step, is more suitable for long-term weather prediction than CNN (direct). However, CNN (iterative) usually requires more iterative steps to obtain an accurate prediction value, the training time and computational burden of the model are more significant, and the training process easily falls into the local optimal solution or requires a unique optimization strategy, which makes the training more difficult.

ConvLSTM combines the temporal modeling capability of LSTM and the spatial feature extraction capability of CNN. It can capture the spatial features in the data and effectively model the dynamic changes in the time series. It is more suitable for handling meteorological data with obvious time evolution patterns. However, ConvLSTM is required to cope with convolutional and recursive operations, leading to long training and inference times on large-scale spatiotemporal datasets, especially when dealing with high dimensions such as meteorological data. By performing horizontal resolution calculations using a spherical harmonic function expansion, the IFS model can accurately handle data from a spherical structure such as the Earth while avoiding boundary problems that tend to arise with many other numerical methods. Also, for T850 prediction, STWPM’s prediction accuracy is always better than the IFS model’s. Moreover, the resolution of the dataset used in STWPM is 5.625 × 5.625, while IFS uses more refined grid point data, which means that our model uses less data to achieve higher prediction accuracy. The above results illustrate that STWPM nicely captures the evolutionary characteristics of complex weather systems, handles the spatial and temporal dependencies of meteorological data well, and is also more suitable for predicting long-time meteorological data series.

#### 3.3.2. Robustness Analysis

**Figure 4:**
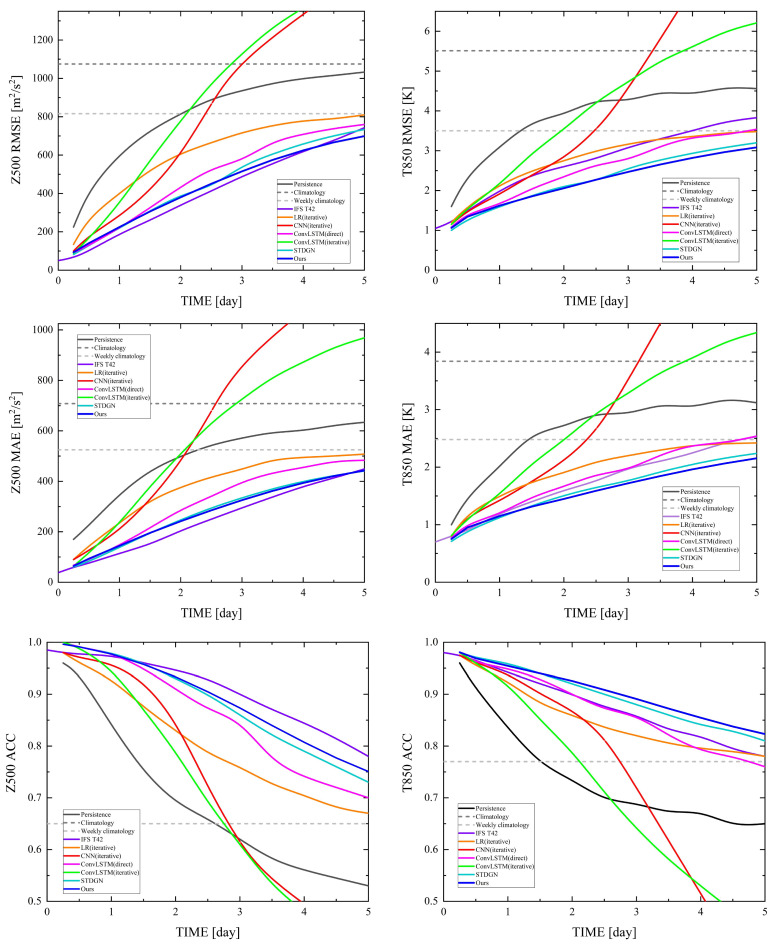
Evaluation of different lead time forecasts for each method.

To demonstrate the model performance and robustness of STWPM in generating multivariate meteorological fields at different lead times, we plotted the predictions within all leads, and the results are shown in Figure 4. Combining Table 1, Table 2 and Table 3, and Figure 4, we can see that STWPM performs better in each precession period on the meteorological field construction work of Z500 and T850. This indicates that STWPM’s temporal capture module can accurately capture the temporal dynamics evolution law and ensure robustness in the time dimension. In comparison to the fundamental CNN and LR models, the STWPM approach offers a more expansive spatiotemporal perspective, realizing a more comprehensive assessment of spatiotemporal attributes and variable coupling. Compared with ConvLSTM and STDGN, the combination of convolution and self-attention can compensate for the shortcomings of convolution and self-attention in extracting spatial features and obtaining adequate spatiotemporal evolution laws. Moreover, the direct prediction method of STWPM can avoid the cumulative error caused by iterative prediction and ensure the stability of medium and long-term prediction. This is verified by the fact that STWPM predictions outperform them more as time is extended in Figure 4. It is worth mentioning that the direct multi-step prediction mechanism of STWPM can train once like an iterative model and then output the future weather data in the short, medium, and long term without the need to train multiple models to achieve temperature predictions for different lead times like other direct prediction models.

To verify the performance of STWPM in predicting regions at different scales, we chose northeast China for localized weather prediction, and Table 4 shows the comparison of the prediction results of each algorithm. Unlike the global scale, the spatial resolution of the northeast China region is 0.25 degrees, and the observation points have a closer connection. Moreover, the complex topography and strong meteorological fluctuations in the northeast region bring great difficulties to the prediction. From the table, it can be seen that STWPM performed the best in the prediction of weather in the northeast region, based on all the three indicators of RMSE, MSE, and ACC. This reflects the robust performance of STWPM in coping with different scale regions, and indicates that STWPM can be applied to meteorological prediction at multiple scales.

#### 3.3.3. Ablation Analysis

To measure the role of the loss function, we launched a loss function ablation study using the proposed model as a baseline. MS in Table 5 denotes the composite loss function of MSE and SSIM. The ablation results indicate that the combined loss function of MSE and SSIM can effectively help the model optimize the fitting effect and improve the prediction accuracy. This is because SSIM helps the model to have a better grasp on the structure of the meteorological distribution map, thus realizing the efficient fitting of the local hotspots and the overall global distribution. In Table 1, Table 2 and Table 3, compared with CNN and self-attentive models, STWPM achieves leading prediction results, which is due to the fact that the network design based on CNN-Transformer can capture the spatio-temporal evolutionary characteristics of meteorology more adequately.

#### 3.3.4. Visualization Analysis

**Figure 5:**
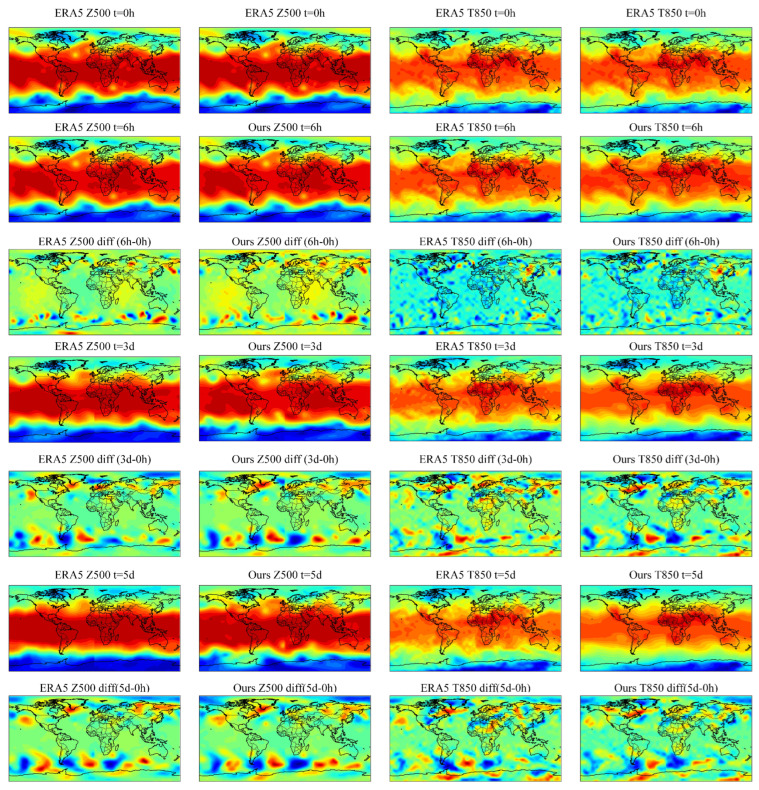
Visualization results for different prediction times.

To visualize STWPM’s spatiotemporal capture capability, we use the STWPM model to predict the Z500 and T850 and compare them with the true values of the ERA5 dataset. The first row is the real value at 0 h, the second row is the predicted value at 6 h, the fourth and sixth rows are the predicted values at three days and five days, respectively, and the third, fifth, and seventh rows are the interpolated values of the corresponding predicted values in the corresponding predicted moments relative to the real value of 0 h. From the error analysis of the third, fifth, and seventh rows of Figure 5 with the real value of 0 h, it can be seen that the model predicts Z500 better than T850, which is because the altitude of Z500 belongs to the middle and upper troposphere, where the atmosphere is more stable and is less affected by the near-surface turbulence, radiative cooling, and other micro-processes, and thus the evolution process is relatively smooth. Conversely, the altitude of T850 belongs to the lower troposphere, and the temperature field in this region is strongly influenced by the surface, especially in the vicinity of complex terrain such as mountains and oceans, where the temperature varies rapidly and with significant spatial differences. The solar radiation heating during the daytime and the turbulence within the boundary layer affect the T850, which complicates the temporal and spatial variability of the T850. The STWPM can effectively capture the global and local variations of meteorological elements, and the prediction effect is more accurate, allowing a better prediction of the actual variations in meteorological elements. The forecast with a lead time of 5 days shows greater prediction accuracy, indicating that the model has better long-term prediction ability.

#### 3.3.5. Efficiency Analysis

To demonstrate that our approach has higher algorithmic efficiency, we designed algorithmic inference time comparison experiments on the same computer platform. Using an iterative approach to predict the weather field content for a 5-day lead time, CNN took 0.5 s, and ConvLSTM took 1.41 s. Because the networks of both models are more straightforward, the inference is faster, but they struggle to meet accuracy requirements. The IFS T42 model has the highest accuracy among the numerical models, but its single inference time reaches 275 s. The STGAN model spends 1.08 s to perform a single task, 254.63 times less than the IFS. The STWPM model takes only 15 milliseconds to perform a single prediction task with a lead time of 5 days, which is 72 times faster than STGAN. Therefore, STWPM has higher computational efficiency with guaranteed prediction accuracy compared with other deep learning models.

## 4. Conclusions

Accurate weather prediction is of great significance for human production and life, natural environmental protection, and extreme weather prediction, while most of the existing data-driven weather prediction models based on multi-point prediction or single spatiotemporal field prediction methods struggle to meet the actual needs of weather prediction. In this paper, we forecast global weather data from the perspective of multivariate spatiotemporal fields using in-analyzed data from ERA5. We developed a spatiotemporal coupled prediction model, STWPM, based on convolution and self-attention, which allows our model to directly output all the weather data in the future for a set lifting period, avoiding the iterative errors caused by iterative prediction methods and the multi-modeling scheme of general direct prediction methods. Among them, to realize full attention to spatial information, we proposed a self-attention-based spatial encoder-decoder to obtain more comprehensive weather feature information. After that, we design a multi-scale spatiotemporal evolution module, achieving the capture of multivariate dynamic coupling relationships and the variable time evolution law through the capture of intra- and inter-frame features. To make our model better focus on the weather change characteristics in the fluctuating region, we design a composite loss function based on MSE and SSIM, which guides our model to fit the dynamic evolution of weather through global and structural losses. Through these careful designs, STWPM can capture more comprehensive multivariate spatiotemporal features and achieve more accurate weather forecasts. Experiments show that STWPM performs better than state-of-the-art methods in both 3-day and 5-day lead time forecasts. Based on this, we believe that the practical application of STWPM in weather prediction will perform better if we continue to explore optimization schemes for more variables in our subsequent work.

## Figures and Tables

**Table 1:** RMSE for 3/5-day lead-time experiments in global regions with different algorithms.

Model	3-Day		5-Day	
	Z500 [m^2^/s^2^]	T850 [K]	Z500 [m^2^/s^2^]	T850 [K]
Persistence	936	4.23	1033	4.56
Climatology	1075	5.51	1075	5.51
Weekly climatology	816	3.5	816	3.5
Linear regression (direct)	693	3.19	783	3.44
Linear regression (iterative)	718	3.17	810	3.48
CNN (direct)	626	2.87	757	3.37
CNN (iterative)	1114	4.48	1559	9.69
ConvLSTM (direct)	570.9	2.76	759.78	3.54
ConvLSTM (iterative)	1128.52	7.42	1525.54	6.21
IFS T42	489	3.09	743	3.83
STDGN	545.72	2.66	736.62	3.29
STWPM	515.14	2.47	699.25	3.08

**Table 2:** MAE for 3/5-day lead-time experiments in global regions with various algorithms.

Model	3-Day		5-Day	
	Z500 [m^2^/s^2^]	T850 [K]	Z500 [m^2^/s^2^]	T850 [K]
Persistence	572	2.91	634	3.12
Climatology	708	3.84	708	3.84
Weekly climatology	525	2.48	525	2.48
Linear regression (direct)	431	2.23	489	2.41
Linear regression (iterative)	447	2.2	580	2.42
CNN (direct)	430	2.02	476	2.38
CNN (iterative)	892	3.49	1263	7.49
ConvLSTM (direct)	398.12	1.95	483.51	2.54
ConvLSTM (iterative)	725.67	3.28	968.87	4.34
IFS T42	295	1.99	449	2.53
STDGN	334.45	1.9	441.15	2.33
STWPM	352.29	1.72	442.81	2.15

**Table 3:** ACC for 3/5-day lead-time experiments in global regions with various algorithms.

Model	3-Day		5-Day	
	Z500	T850	Z500	T850
Persistence	0.62	0.69	0.53	0.65
Climatology	0	0	0	0
Weekly climatology	0.65	0.77	0.65	0.77
Linear regression (direct)	0.76	0.81	0.68	0.78
Linear regression (iterative)	0.76	0.82	0.67	0.78
CNN (direct)	0.81	0.85	0.71	0.79
CNN (iterative)	0.61	0.72	0.41	0.31
ConvLSTM (direct)	0.85	0.86	0.7	0.76
ConvLSTM (iterative)	0.61	0.64	0.39	0.45
IFS T42	0.9	0.86	0.78	0.8
STDGN	0.86	0.87	0.73	0.8
STWPM	0.87	0.89	0.75	0.82

**Table 4:** Comparison results of the prediction of different algorithms in small-scale regions.

Projects		3-Day		5-Day	
		Z500 [m^2^/s^2^]	T850 [K]	Z500 [m^2^/s^2^]	T850 [K]
RMSE	CNN	1162.44	7.54	1663.98	9.59
	ConvLSTM	887.09	4.69	1074.47	5.72
	STDGN	820.17	4.39	925.32	5.19
	STWPM	795.76	4.24	860.14	4.58
MAE	CNN	1011.74	6.62	1488.44	8.50
	ConvLSTM	759.65	3.91	911.98	4.72
	STDGN	715.02	3.73	820.17	4.52
	STWPM	696.77	3.61	757.39	3.93
ACC	CNN	0.83	0.85	0.71	0.72
	ConvLSTM	0.87	0.92	0.81	0.88
	STDGN	0.90	0.93	0.87	0.90
	STWPM	0.89	0.93	0.88	0.92

**Table 5:** Ablation result of the loss function.

Projects		3-Day		5-Day	
		Z500 [m^2^/s^2^]	T850 [K]	Z500 [m^2^/s^2^]	T850 [K]
RMSE	STWPM w/o MS	811.1	4.33	872.82	4.7
	STWPM	795.76	4.24	860.14	4.58
MAE	STDGN w/o MS	711.05	3.71	769.3	4.06
	STWPM	696.77	3.61	757.39	3.93
ACC	STWPM w/o MS	0.89	0.93	0.87	0.91
	STWPM	0.89	0.93	0.88	0.92

## Data Availability

Data will be made available on request.

## Appendix A

The Structural Similarity Index (SSIM) is a metric used to measure the similarity between two images, commonly applied in image quality assessment. The formula for SSIM is as follows:

$$SSIM\left(x,y\right)=\frac{\left(2\mu_{x}\mu_{y}+C_{1}\right)\left(2\sigma_{xy}+C_{2}\right)}{\left(\mu_{x}^{2}+\mu_{y}^{2}+C_{1}\right)\left(\sigma_{x}^{2}+\sigma_{y}^{2}+C_{2}\right)}$$

where:

*x*, *y*: represent the ground truth image and the predicted image, respectively.
$\mu_{x}$, $\mu_{y}$: the mean values of *x* and *y*, representing the luminance of the images.
$\sigma_{x}^{2}$, $\sigma_{y}^{2}$: the variances of *x* and *y*, representing the contrast of the images.
$\sigma_{xy}$: the covariance of *x* and *y*, representing the structural similarity between the images.
$C_{1}={\left(K_{1}\cdot L\right)}^{2}$, $C_{2}={\left(K_{2}\cdot L\right)}^{2}$: small constants included to stabilize the calculation. Here, *L* is the dynamic range of the values, and $K_{1}$ and $K_{2}$ are constants, typically set to $K_{1}=0.01$ and $K_{2}=0.03$.
SSIM values range from −1 to 1, where a value closer to 1 indicates greater similarity between the two images.

## References

[1] Li X., Feng M., Ran Y., Su Y., Liu F., Huang C., Shen H., Xiao Q., Su J., Yuan S. (2023). Big Data in Earth system science and progress towards a digital twin. Nat. Rev. Earth Environ..

[2] Smith D.M., Eade R., Scaife A.A., Caron L.P., Danabasoglu G., DelSole T.M., Delworth T., Doblas-Reyes F.J., Dunstone N.J., Hermanson L. (2019). Robust skill of decadal climate predictions. NPJ Clim. Atmos. Sci..

[3] Hao Z., Singh V.P., Xia Y. (2018). Seasonal drought prediction: Advances, challenges, and future prospects. Rev. Geophys..

[4] Yeager S.G., Danabasoglu G., Rosenbloom N.A., Strand W., Bates S.C., Meehl G.A., Karspeck A.R., Lindsay K., Long M.C., Teng H. (2018). Predicting near-term changes in the earth system: A large ensemble of initialized decadal prediction simulations using the community earth system model. Bull. Am. Meteorol. Soc..

[5] Chevuturi A., Turner A.G., Johnson S., Weisheimer A., Shonk J.K., Stockdale T.N., Senan R. (2021). Forecast skill of the Indian monsoon and its onset in the ECMWF seasonal forecasting system 5 (SEAS5). Clim. Dyn..

[6] Elahi E., Zhu M., Khalid Z., Wei K. (2024). An empirical analysis of carbon emission efficiency in food production across the Yangtze River basin: Towards sustainable agricultural development and carbon neutrality. Agric. Syst..

[7] Abbas A., Zhao C., Ullah W., Ahmad R., Waseem M., Zhu J. (2021). Towards Sustainable Farm Production System: A Case Study of Corn Farming. Sustainability.

[8] Ma Z., Liu B., Mehra A., Abdolali A., van der Westhuysen A., Moghimi S., Vinogradov S., Zhang Z., Zhu L., Wu K. (2020). Investigating the impact of high-resolution land–sea masks on hurricane forecasts in HWRF. Atmosphere.

[9] Bauer P., Thorpe A., Brunet G. (2015). The quiet revolution of numerical weather prediction. Nature.

[10] Wu Y., Xue W. (2024). Data-Driven Weather Forecasting and Climate Modeling from the Perspective of Development. Atmosphere.

[11] Miyachi T., Enomoto T. (2021). Tropical cyclone track forecasts using NCEP-GFS with initial conditions from three analyses. SOLA.

[12] Fleury A., Bouttier F., Couvreux F. (2022). Process-oriented stochastic perturbations applied to the parametrization of turbulence and shallow convection for ensemble prediction. Q. J. R. Meteorol. Soc..

[13] Zhang Y., Long M., Chen K., Xing L., Jin R., Jordan M.I., Wang J. (2023). Skilful nowcasting of extreme precipitation with NowcastNet. Nature.

[14] Sachindra D.A., Ahmed K., Rashid M.M., Shahid S., Perera B.J.C. (2018). Statistical downscaling of precipitation using machine learning techniques. Atmos. Res..

[15] Wang Z., Lai C., Chen X., Yang B., Zhao S., Bai X. (2015). Flood hazard risk assessment model based on random forest. J. Hydrol..

[16] Zhang T., Liang Z., Wang H., Wang J., Hu Y., Li B. (2023). Merging multisatellite precipitation products using stacking method and the censored-shifted gamma ensemble model output statistics in china’s Beimiaoji basin. J. Hydrol..

[17] Chiang Y.M., Hao R.N., Zhang J.Q., Lin Y.T., Tsai W.P. (2018). Identifying the sensitivity of ensemble streamflow prediction by artificial intelligence. Water.

[18] Pirone D., Cimorelli L., Del Giudice G., Pianese D. (2023). Short-term rainfall forecasting using cumulative precipitation fields from station data: A probabilistic machine learning approach. J. Hydrol..

[19] Khan N., Sachindra D.A., Shahid S., Ahmed K., Shiru M.S., Nawaz N. (2020). Prediction of droughts over Pakistan using machine learning algorithms. Adv. Water Resour..

[20] Di Nunno F., Zhu S., Ptak M., Sojka M., Granata F. (2023). A stacked machine learning model for multi-step ahead prediction of lake surface water temperature. Sci. Total Environ..

[21] Alelyani S. (2021). Detection and evaluation of machine learning bias. Appl. Sci..

[22] Shang Y., Guo J., Wu W. (2019). Machine learning methods embedded with domain knowledge (part ii): Generalization risk. Proc. CSEE.

[23] Chen J., Zeng G.Q., Zhou W., Du W., Lu K.D. (2018). Wind speed forecasting using nonlinear-learning ensemble of deep learning time series prediction and extremal optimization. Energy Convers. Manag..

[24] Frame J.M., Kratzert F., Klotz D., Gauch M., Shalev G., Gilon O., Qualls L.M., Gupta H.V., Nearing G.S. (2022). Deep learning rainfall–runoff predictions of extreme events. Hydrol. Earth Syst. Sci..

[25] Xiang Z., Yan J., Demir I. (2022). A rainfall-runoff model with LSTM-based sequence-to-sequence learning. Water Resour. Res..

[26] Vo T.Q., Kim S.H., Nguyen D.H., Bae D.H. (2023). LSTM-CM: A hybrid approach for natural drought prediction based on deep learning and climate models. Stoch. Environ. Res. Risk Assess..

[27] Ni L., Wang D., Singh V.P., Wu J., Wang Y., Tao Y., Zhang J. (2020). Streamflow and rainfall forecasting by two long short-term memory-based models. J. Hydrol..

[28] Shi X., Chen Z., Wang H., Yeung D.Y., Wong W.K., Woo W.C. Convolutional LSTM network: A machine learning approach for precipitation nowcasting. Proceedings of the Annual Conference on Neural Information Processing Systems 2015.

[29] Hao P., Li S., Song J., Gao Y. (2023). Prediction of sea surface temperature in the South China Sea based on deep learning. Remote Sens..

[30] Dehghani A., Moazam H.M.Z.H., Mortazavizadeh F., Ranjbar V., Mirzaei M., Mortezavi S., Ng J.L., Dehghani A. (2023). Comparative evaluation of LSTM, CNN, and ConvLSTM for hourly short-term streamflow forecasting using deep learning approaches. Ecol. Inform..

[31] Uluocak I., Bilgili M. (2024). Daily air temperature forecasting using LSTM-CNN and GRU-CNN models. Acta Geophys..

[32] Luo W., Li Y., Urtasun R., Zemel R. Understanding the effective receptive field in deep convolutional neural networks. Proceedings of the Annual Conference on Neural Information Processing Systems 2016.

[33] Zamir S.W., Arora A., Khan S., Hayat M., Khan F.S., Yang M.H. Restormer: Efficient transformer for high-resolution image restoration. Proceedings of the IEEE/CVF Conference on Computer Vision and Pattern Recognition (CVPR).

[34] Zeiler M.D., Fergus R. (2014). Visualizing and understanding convolutional networks. Proceedings of the 13th European Conference on Computer Vision—ECCV 2014.

[35] Lin Z., Feng M., Santos C.N.D., Yu M., Xiang B., Zhou B., Bengio Y. (2017). A structured self-attentive sentence embedding. arXiv.

[36] Devlin J. (2018). Bert: Pre-training of deep bidirectional transformers for language understanding. arXiv.

[37] Vaswani A. Attention is all you need. Proceedings of the 31st Conference on Neural Information Processing Systems (NIPS 2017).

[38] Radford A. (2018). Improving Language Understanding by Generative Pre-Training. https://openai.com/index/language-unsupervised/.

[39] Gulati A., Qin J., Chiu C., Parmar N., Zhang Y., Yu J., Han W., Wang S., Zhang Z., Wu Y. (2020). Conformer: Convolution-augmented transformer for speech recognition. arXiv.

[40] Parisotto E., Salakhutdinov R. (2021). Efficient transformers in reinforcement learning using actor-learner distillation. arXiv.

[41] Dosovitskiy A. (2020). An image is worth 16 × 16 words: Transformers for image recognition at scale. arXiv.

[42] Lin L., Zhang Z., Yu H., Wang J., Gao S., Zhao H., Zhang J. (2024). StHCFormer: A Multivariate Ocean Weather Predicting Method Based on Spatiotemporal Hybrid Convolutional Attention Networks. IEEE J. Sel. Top. Appl. Earth Obs. Remote Sens..

[43] Child R., Gray S., Radford A., Sutskever I. (2019). Generating long sequences with sparse transformers. arXiv.

[44] Cordonnier J.B., Loukas A., Jaggi M. (2019). On the relationship between self-attention and convolutional layers. arXiv.

[45] Hersbach H., Bell B., Berrisford P., Hirahara S., Horányi A., Muñoz-Sabater J., Nicolas J., Peubey C., Radu R., Schepers D. (2020). The ERA5 global reanalysis. Q. J. R. Meteorol. Soc..

[46] Zhang Z., Lin L., Gao S., Wang J., Zhao H. (2024). Wind speed prediction in China with fully-convolutional deep neural network. Renew. Sustain. Energy Rev..

[47] Zhang J., Wang B., Hua M., Chen Z., Liang S., Kang X. (2024). Spatiotemporal Meteorological Prediction Based on Fully Convolutional Neural Network. IEEE Trans. Geosci. Remote Sens..

[48] Rasp S., Dueben P.D., Scher S., Weyn J.A., Mouatadid S., Thuerey N. (2020). WeatherBench: A benchmark data set for data-driven weather forecasting. J. Adv. Model. Earth Syst..

